# Relationship between consciousness level and perfusion computed tomography in patients with prolonged disorders of consciousness

**DOI:** 10.18632/aging.204417

**Published:** 2022-12-05

**Authors:** Qi Xiong, Yong Wang, Ziwen Wang, Yunliang Tang, Lianghua Huang, Junwei Kang, Zhen Feng

**Affiliations:** 1 Department of Rehabilitation Medicine, The First Affiliated Hospital of Nanchang University, Nanchang 330006, Jiangxi, P.R. China; 2 Department of Medical Oncology, The First Affiliated Hospital of Nanchang University, Nanchang 330006, Jiangxi, P.R. China

**Keywords:** disorder of consciousness, whole brain perfusion CT, conscious impairment, vegetative state, minimally conscious state

## Abstract

Purpose: We assessed the relationship between consciousness level and values of cerebral blood flow (CBF), cerebral blood volume (CBV), mean transit time (MTT), and time to peak (TTP) obtained by whole-brain perfusion computed tomography (pCT) in patients with prolonged disorders of consciousness (pDOC).

Methods: This study included 29 patients in vegetative state (VS), 34 with minimally consciousness state minus (MCS−), and 13 with minimally consciousness state plus (MCS+). All patients were evaluated using the Coma Recovery Scale-Revised (CRS-R), the Glasgow Coma Scale (GCS), and the Full Outline of UnResponsiveness (FOUR). The values of CBF, CBV, MTT, and TTP were obtained from patients who underwent pCT. Differences in CBF, CBV, MTT, and TTP were compared between the three types of pDOC. Correlations between the CRS-R, GCS, and FOUR scores and the pCT results were analyzed.

Results: Among the three groups, patients in VS showed a significantly decreased CBF in the bilateral frontal lobe, thalamus, temporal lobe, occipital lobe, brainstem, and damaged part. CBV was significantly reduced in patients with VS in the bilateral frontal lobe, thalamus, temporal lobe, brainstem, and damaged part. The total CRS-R, GCS, and FOUR scores were positively correlated with CBF, CBV, and TTP in almost all regions of interest.

Conclusion: Reductions in CBF and CBV calculated with pCT are associated with impaired consciousness and perfusion CT could be a promising tool in evaluating the conscious level in patients with pDOC.

## INTRODUCTION

Disorders of consciousness (DOC) are characterized by prolonged impaired consciousness following severe acquired brain injuries or nervous system dysfunction (e.g., heart or lung failure, cardiac arrest etc). Prolonged disorder of consciousness (pDOC) are defined by a coma condition usually lasting more than four weeks after severe injury [1]. A pDOC is further subcategorized as vegetative state (VS), minimally conscious state minus (MCS−), and minimally conscious state plus (MCS+) [2]. Vegetative state/unresponsive wakefulness syndrome is a condition in which patients open their eyes but show no clinical evidence of consciousness [3]. The most frequent signs of consciousness in MCS minus patients are visual fixation and pursuit, automatic motor reactions (e.g., scratching, pulling the bed sheet) and localization to noxious stimulation, whereas MCS plus patients can, in addition, follow simple commands, intelligibly verbalize or intentionally communicate [4]. Patients with pDOC are often associated with high mortality, high rates of complications, and high medical costs, and the differential diagnosis of pDOC has remained an issue for years. A Previous study have shown that 41% of patients diagnosed with VS demonstrated signs of awareness [5]. In recent years, neuroimaging has become a widely useful clinical tool for pDOC patients. Computed tomography (CT) and magnetic resonance (MR) are widely used to evaluate the degree of brain damage and detect organic causes of pDOC. New imaging techniques such as MR spectroscopy (MRS), single-photon emission computed tomography (SPECT), functional MR imaging (fMRI), positron emission tomography (PET) allow for investigation of metabolism, brain function, activation, and blood perfusion [6, 7]. These techniques have some noticeable deficits, such as high time consumption, high cost and the use of radioactive materials. MRI has the characteristic of being non-invasive [8], but MRI examination takes more time, which limits its application in patients who cannot cooperate, especially patients in minimally conscious state, and only directly obtains an estimate of cerebral blood flow (CBF). However, the utility of neuroimaging in differentiating between vegetative state or unresponsive waking syndromes (VS/UWS) and minimally conscious states (MCS−/+) remains uncertain.

Perfusion computed tomography (PCT) has revolutionized CT imaging, as a cost-effective, quick, easy to perform and low radioactivity technique broadened its applications. Recent studies have found that PCT can be a useful adjunct to traditional CT, helping to define changes in cerebral blood flow (CBF), cerebral blood flow (CBV), mean transit time (MTT), and time to peak (TTP). PCT is used extensively in the evaluation of acute ischemic stroke patients for improved stroke diagnosis, assessment of core infarction and viable but hypo perfused tissue (penumbra) [9, 10], but also for vasospasm, tumors [11], and traumatic brain injury [12]. However, pCT is an imaging method that has not been widely used for pDOC. The Coma Remission Scale-revised (CRS-R) is the golden standard to assess the level of consciousness [13, 14], and Glasgow Coma Scale (GCS) and Full Scale of Unresponsiveness (FOUR) are important and widely used tools to evaluate the degree of damage in brain injury [15, 16]. To our knowledge, no studies concerning the relationship between consciousness level and the results of pCT in pDOC have been reported until now, which limits pCT’s application in pDOC.

The purpose of this study is to assess the relationship between consciousness level and values of cerebral blood flow (CBF), cerebral blood volume (CBV), mean transit time (MTT), and time to peak (TTP) and to compare the differences in these values between three types of pDOC.

## RESULTS

### Characteristics of patients at study entry as a function of diagnosis

**Figure 1:**
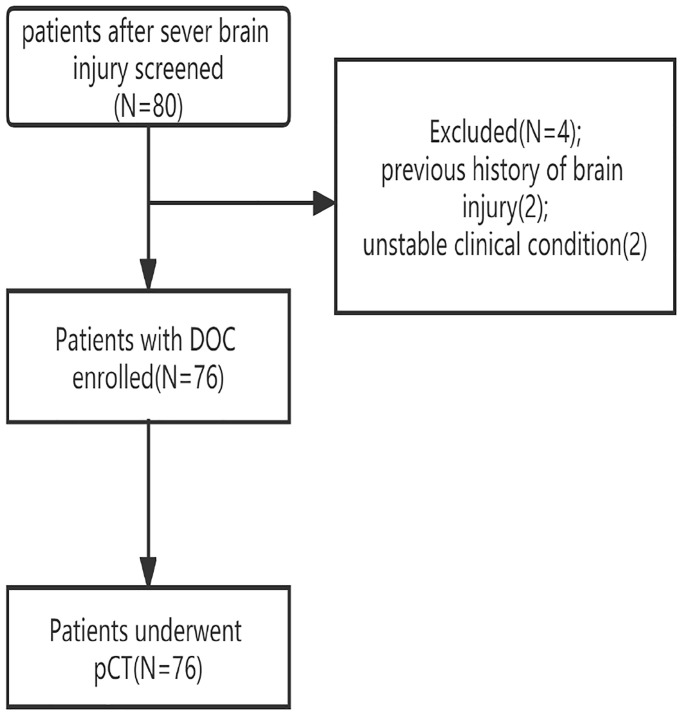
Study flowchart.

Out of 80 patients screened for this study, 76 patients with pDOC (mean age 49.6 ± 14.3, VS 29, MCS− 34, MCS+ 13) fulfilled the selection criteria (Figure 1 study flowchart). Demographic and clinical indices for three diagnosis group are presented in Table 1. No significant differences were observed between the three types of pDOC in age, pDOC duration, time to emergency, gender, job, married, education level, and etiology. Patients with MCS+ had the highest means total scores of Coma Recovery Scale-Revised (CRS-R), Glasgow Coma Scale (GCS), and Full Scale of Unresponsiveness (FOUR) scores.

**Table 1:** Characteristics of patients with prolonged disorders of consciousness.

	**VS**	**MCS−**	**MCS+**	***p* value**
N	29	34	13	
GCS	6.4 ± 2.0	7.4 ± 1.5	11.0 ± 2.0	<0.001
FOUR	8.8 ± 2.4	10.2 ± 1.7	12.4 ± 1.1	<0.001
CRS-R	5.1 ± 2.0	6.5 ± 1.4	8.5 ± 1.4	<0.001
Age (y.o)	53.0 ± 12.6	51.4 ± 18.2	48.6 ± 10.3	0.574
pDOC duration (days)	54.3 ± 36.2	46.5 ± 30.9	45.1 ± 28.2	0.544
Time to emergency (hours)	1.8 ± 2.3	1.2 ± 1.4	1.3 ± 1.3	0.419
Gender (female/male)	6/23	11/23	4/9	0.5650
Job (yes/no)	19/10	19/15	7/6	0.6742
Married (yes/no)	27/2	28/6	12/1	0.3695
Education level (elementary/middle/high)	13/13/3	15/11/8	6/6/1	0.5286
Etiology (TBI/Non-TBI)	12/17	18/16	6/7	0.4977

### Differences in CBF, CBV, MTT, and TTP between the three types of pDOC

**Figure 2:**
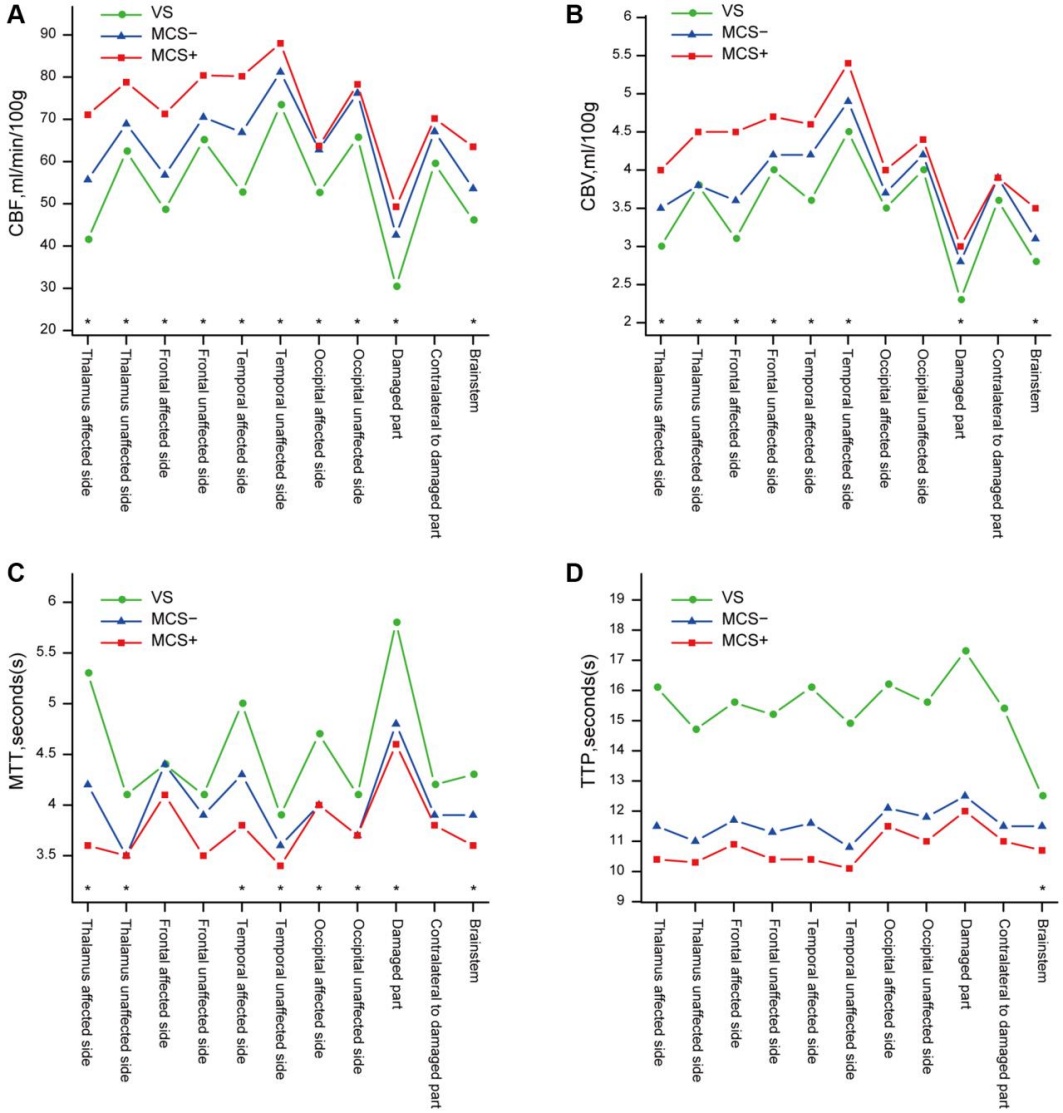
**Differences in the results of pCT between the three types of pDOC.** (**A**) Differences of CBF between the three types of pDOC, (**B**) Differences of CBV, (**C**) Differences of MTT, (**D**) Differences of TTP. ^*^presents a statistically significant difference (*p* < 0.05).

Among the three groups, patients with VS showed a significantly decreased CBF in the bilateral frontal lobe, thalamus, temporal lobe, occipital lobe, brainstem, and damaged part. CBV was significantly decreased in patients with VS in the bilateral frontal lobe, thalamus, temporal lobe, brainstem, and damaged part. The mean MTT significantly differed between the three types of pDOC in the bilateral thalamus, temporal lobe, occipital lobe, brainstem, and damaged part. No significant differences in mean TTP were found between the three types of pDOC, except in the brainstem (Figure 2).

### Relationships between behavioral assessment scores and results of pCT

The total CRS-R, GCS, and FOUR scores were positively correlated with CBF, CBV, and TTP in almost all regions. CRS-R scores were correlated with MTT in almost all regions, and GCS and FOUR scores were correlated with MTT in the frontal lobe, temporal lobe, thalamus, and occipital lobe of the affected side. Correlation analysis of the age, pDOC duration and the CBF, CBV values revealed negative correlations in all regions, but all of them were of no statistical significance. In addition, there was a negative correlation between mean age and TTP (Tables 2–5).

**Table 2:** Correlation of the CBF values and the results of behavioral assessments.

	**Age**	**pDOC duration**	**GCS**	**FOUR**	**CRS-R**
Thalamus affected side	−0.13	−0.15	0.44^*^	0.37^*^	0.39^*^
Thalamus unaffected side	−0.14	−0.03	0.55^*^	0.44^*^	0.46^*^
Frontal affected side	−0.27	−0.08	0.69^*^	0.69^*^	0.52^*^
Frontal unaffected side	−0.18	−0.11	0.53^*^	0.40^*^	0.35^*^
Temporal affected side	−0.21	−0.03	0.43^*^	0.40^*^	0.34^*^
Temporal unaffected side	−0.03	−0.01	0.38^*^	0.31^*^	0.32^*^
Occipital affected side	−0.02	−0.13	0.38^*^	0.38^*^	0.26^*^
Occipital unaffected side	−0.01	−0.21	0.31^*^	0.18	0.21
Damaged part	−0.13	−0.03	0.47^*^	0.48^*^	0.38^*^
Contralateral to damaged part	−0.07	−0.07	0.38^*^	0.27^*^	0.35^*^
Brainstem	−0.12	−0.11	0.54^*^	0.52^*^	0.42^*^

**Table 3:** Correlation of the CBV values and the results of behavioral assessments.

	**Age**	**pDOC duration**	**GCS**	**FOUR**	**CRS-R**
Thalamus affected side	−0.23	−0.10	0.41^*^	0.28^*^	0.27^*^
Thalamus unaffected side	−0.11	0.07	0.49^*^	0.36^*^	0.41^*^
Frontal affected side	−0.25^*^	−0.09	0.49^*^	0.43^*^	0.32^*^
Frontal unaffected side	−0.24^*^	−0.06	0.42^*^	0.32^*^	0.22
Temporal affected side	−0.16	−0.01	0.35^*^	0.28^*^	0.22^*^
Temporal unaffected side	−0.10	−0.06	0.37^*^	0.35^*^	0.30^*^
Occipital affected side	−0.06	−0.17	0.24^*^	0.18	0.11^*^
Occipital unaffected side	−0.07	0.14	0.26	0.12^*^	0.68
Damaged part	−0.09	−0.07	0.31^*^	0.29^*^	0.19^*^
Contralateral to damaged part	−0.10	−0.03	0.29^*^	0.15	0.24^*^
Brainstem	−0.05	−0.16	0.51^*^	0.44^*^	0.38^*^

**Table 4:** Correlation of the MTT values and the results of behavioral assessments.

	**Age**	**pDOC duration**	**GCS**	**FOUR**	**CRS-R**
Thalamus affected side	0.07	0.14	−0.17	−0.18	−0.24^*^
Thalamus unaffected side	0.02	0.06	−0.19	−0.19	−0.23^*^
Frontal affected side	0.07	0.03	−0.23^*^	−0.28^*^	−0.20
Frontal unaffected side	0.12	0.02	−0.23	−0.30	−0.27^*^
Temporal affected side	0.16	0.11	−0.25^*^	−0.31^*^	−0.29^*^
Temporal unaffected side	0.01	0.08	−0.19	−0.15	−0.24^*^
Occipital affected side	0.11	0.02	−0.23^*^	−0.31^*^	−0.26^*^
Occipital unaffected side	0.04	0.11	−0.10	−0.10	−0.21
Damaged part	0.14	0.07	−0.44^*^	−0.42^*^	−0.42^*^
Contralateral to damaged part	0.45	0.09	−0.31^*^	−0.30^*^	−0.31^*^
Brainstem	0.09	0.35	−0.29^*^	−0.32^*^	−0.25^*^

**Table 5:** Correlation of the TTP values and the results of behavioral assessments.

	**Age**	**pDOC duration**	**GCS**	**FOUR**	**CRS-R**
Thalamus affected side	0.27^*^	0.17	−0.31^*^	−0.28^*^	−0.36^*^
Thalamus unaffected side	0.34^*^	0.09	−0.32^*^	−0.34^*^	−0.33^*^
Frontal affected side	0.37^*^	0.05	−0.29^*^	−0.35^*^	−0.33^*^
Frontal unaffected side	0.37^*^	0.14	−0.37^*^	−0.37^*^	−0.39^*^
Temporal affected side	0.44^*^	0.06	−0.41^*^	−0.45^*^	−0.43^*^
Temporal unaffected side	0.33^*^	0.09	−0.36^*^	−0.34^*^	−0.41^*^
Occipital affected side	0.47^*^	0.09	−0.39^*^	−0.43^*^	−0.35^*^
Occipital unaffected side	0.36^*^	0.13	−0.36^*^	−0.39^*^	−0.36^*^
Damaged part	0.28^*^	0.08	−0.37^*^	−0.40^*^	−0.42^*^
Contralateral to damaged part	0.15	0.15	−0.38^*^	−0.39^*^	−0.43^*^
Brainstem	0.34^*^	0.24	−0.42^*^	−0.48^*^	−0.44^*^

## DISCUSSION

Perfusion CT with iodinated contrast medium is a very promising technique for measuring cerebral blood perfusion with several advantages compared to other functional imaging techniques, such as PET, SPECT, fMRI, or MRS [17, 18]. First, pCT is cost-effective, quick, easy to perform, and is widely available on most clinical units. Second, it allows for the assessment of more than one perfusion parameter and obtains quantitative data, including CBF, CBV, and MTT, as well as permeability surface, which is used in tumor diagnostics, and TTP, which is used in the diagnosis of cerebral ischemic diseases. In addition, pCT can be performed without sedation or general anesthesia with a short scanning time (50 s), which is very important in patients with pDOC, who are often uncooperative, especially those with MCS. PCT was shown as a reliable tool for the diagnosis of dementia [19, 20]. However, this two studies by Osawa and Zimny found that CBF were related to the degree of cognitive impairment assessed by mini mental state examination in patients with dementia. To the best of our knowledge, the application of perfusion CT in patients with disorders of consciousness is very rare. No studies has been reported concerning the relationship between the consciousness level and perfusion CT parameters (including CBV, CBF, MTT, TTP) and the differences in those values between different types of pDOC.

Behavioral assessments, including the CRS-R, GCS, and FOUR, are practical methods for determining the level of consciousness of patients with pDOC and are widely used in clinical practice [14–16]. The CRS-R is an excellent and widely used tool to differentiate the diagnosis of VS and MCS [13]. Previous studies have demonstrated the differences between VS and MCS with respect to metabolism using SPECT or PET. A PET study by Stender et al. showed that metabolic differences between VS and MCS were most obvious in the frontoparietal cortex and found PET to be a useful tool in distinguishing MCS from VS/UWS [21].

In our study, patients with VS showed a significant decrease in CBF and CBV in most brain regions, which is similarly to the results of the pCT study by Cooper, in which the CBF and CBV of patients with severe traumatic brain injury were lower than in those with moderate traumatic brain injury [22]. This demonstrates that patients with VS have a poorer blood supply after brain injuries, which could affect the neuroplasticity and prognosis. It is observed that only in the brainstem, significant differences in mean TTP were found between the three types of pDOC. One cause of disorder of consciousness is the damage of ascending reticular activating system of the brainstem, especially the brain stem nerve fiber bundle. The degree of damage to the brainstem nerve fiber is related to level of consciousness [23]. But our study showed that TTP values of the brainstem are significantly different between three types of pDOC, which may indicates that the level of consciousness is not only related to the nerve fiber damage but also to the vascular spasm. We also found that the GCS, FOUR, and CRS-R scores were positively correlated with CBF, CBV, and TTP in almost all regions. This is in line with a pCT study by Trofimov et al., which found that the early GCS scores were positively associated with CBF and CBV in patients with traumatic brain injury [24]. Furthermore, a stronger correlation was found between CBF and CRS-R than between CRS-R and CBV or TTP. This indicates that CBF are better related to conscious level and more suitable for evaluating the level of consciousness. On behavioral assessments, patients with MCS+ had the highest mean CRS-R, GCS, and FOUR scores. In addition, there was a negative correlation between mean age and TTP, which is supported by a study which revealed old age was a risk factor of cerebrovascular stenosis [25].

Although the CRS-R is well known as the most robust and reliable scale for assessing the level of consciousness [13, 14]. Indeed, if pDOC could be earlier and more accurately diagnosed only by clinical features including behavioral assessments, costly methods such as SPECT, PET, and fMRI would have little to add. In fact, with the diagnostic difficulties and limitations of the CRS-R, the diagnostic value of pCT has been enhanced. Thus, we found that a reduction of CBF and CBV detected by pCT agreed with the clinical features of three types of pDOC. The encouraging results of studies concerning the role of pCT in assessing the level of consciousness in patients with pDOC make this technique a promising tool for evaluating the conscious level in patients with pDOC, although more research is needed.

There are several limitations in this study. First, the sample size is small, especially the number of patients in minimally conscious state plus (MCS+). Considering the small sample size, we were unable to analyze the relationship between pCT and level of consciousness between different groups. The correlation coefficients are mostly between 0.3 and 0.69, which may limit its application. The reasons may be the sample size of patients with high CRS-R scores is not large enough, which may cause selection bias and reduce the correlation coefficient. Second, it is better to assess the behavioral score not only on the day of CT, but on consecutive days before and after the examination, especially the CRS-R score. Because a patient’s level of consciousness fluctuates, and only a day’s CRS-R score may not accurately reflect the level of consciousness.

## CONCLUSIONS

The reduction in CBF and CBV calculated by pCT was associated with the level of consciousness in patients with pDOC; Thus, pCT could be a promising tool in evaluating the level of consciousness in patients with pDOC, although further research is necessary. As pCT is a widely available, cost-effective, and simple method of assessing the level of consciousness in patients with pDOC, it can be competitive with other more sophisticated and expensive modalities such as SPECT, PET, fMRI, and MRS.

## METHODS

### Inclusion and exclusion criteria and sample size

For this study, we screened the patients with DOC who were consecutively admitted to a neurorehabilitation unit. The inclusion criteria were as follows: (1) clinical diagnosis of VS or MCS, according to the standard criteria; (2) age ≥18 years; (3) traumatic, vascular, or anoxic etiology; and (4) duration of DOC is longer than 4 weeks. The exclusion criteria were as follows: (1) nervous system dysfunction or unstable clinical condition (e.g., severe heart or respiratory failure); and (2) previous history of brain injury. The diagnosis of patients with pDOC were determined according to the standard diagnostic criteria [26, 27]. The level of consciousness was evaluated by means of the total scores of the CRS-R, GCS, and FOUR, which were assessed by two skilled clinicians both blinded to the results of pCT.

According to the Sample Size Estimation formula for Coefficient Tests [28]:


$$\text{n}=\frac{{\left({\text{Z}}_{1-\text{α}/\text{s}}+{\text{Z}}_{1-\text{β}}\right)}^{2}}{{\left[\text{FZ}({\text{ρ}}_{1})-\text{FZ}({\text{ρ}}_{0})\right]}^{2}}+3$$

FZ is Fisher’s Z transformation’s is two or one side test, ρ_1_ and ρ_0_ are correlation coefficients of null hypothesis and alternative hypothesis.


$$\text{FZ}(\text{ρ})=\frac{1}{2}\text{ln}\left[\frac{1+\text{ρ}}{1-\text{ρ}}\right]$$

The sample size calculation is based on the correlations between behavioral scores and pCT results. We hypothesize that the correlation coefficients are higher than 0.3, which is the lower limitation of poor correlation. In addition, correlation coefficients between CRS-R and CBF in frontal lobe of our pilot trials was 0.52. Thus, with a type I error of 5% (α = 0.05), 80% power (β = 0.20), s = 2, ρ_1_ = 0.52, ρ_0_ = 0.3, the estimated required sample size is 80 in total.

### Behavioral assessments

The CRS-R has 23 items with 6 subscales, including visual, auditory, motor, oromotor, communication, and arousal functions [14]. The higher the total scores, the higher the level of consciousness. The GCS includes 15 items grouped into 3 subscales representing motor, eye, and verbal response [15]. The total GCS score determines the level of consciousness. The FOUR consists of four subscales addressing eye and motor response, brainstem reflexes, and respiratory pattern [16]. The FOUR scores range from 0 to 16, with higher scores indicating higher levels of consciousness.

### Perfusion CT

A dual-source CT scanner (SOMATOM Definition; Siemens Healthineers, Erlangen, Germany) was used to scan all patients with one-stop perfusion CT scanning models. A non-contrast CT scan was first performed with a voltage of 120 kVp and a current-time of 390 mAs. Each patient received an intravenous injection of 60 mL of iopamidol (370 mg iodine/mL; Beilu, Beijing, China) at a rate of 4 mL/L with an automatic injector and 30 mL of a contrast–saline mixture injection received at the same flow rate. pCT images covered the range from the base of the skull to the top of the head starting at contrast injection with the following parameters: voltage, 80 kV; tube current-time, 120 mAs cross-section collimation, 32 × 1.2 mm. pCT images were reconstructed using an H30f convolution kernel with a 3-mm slice thickness in 3 mm increments.

**Figure 3:**
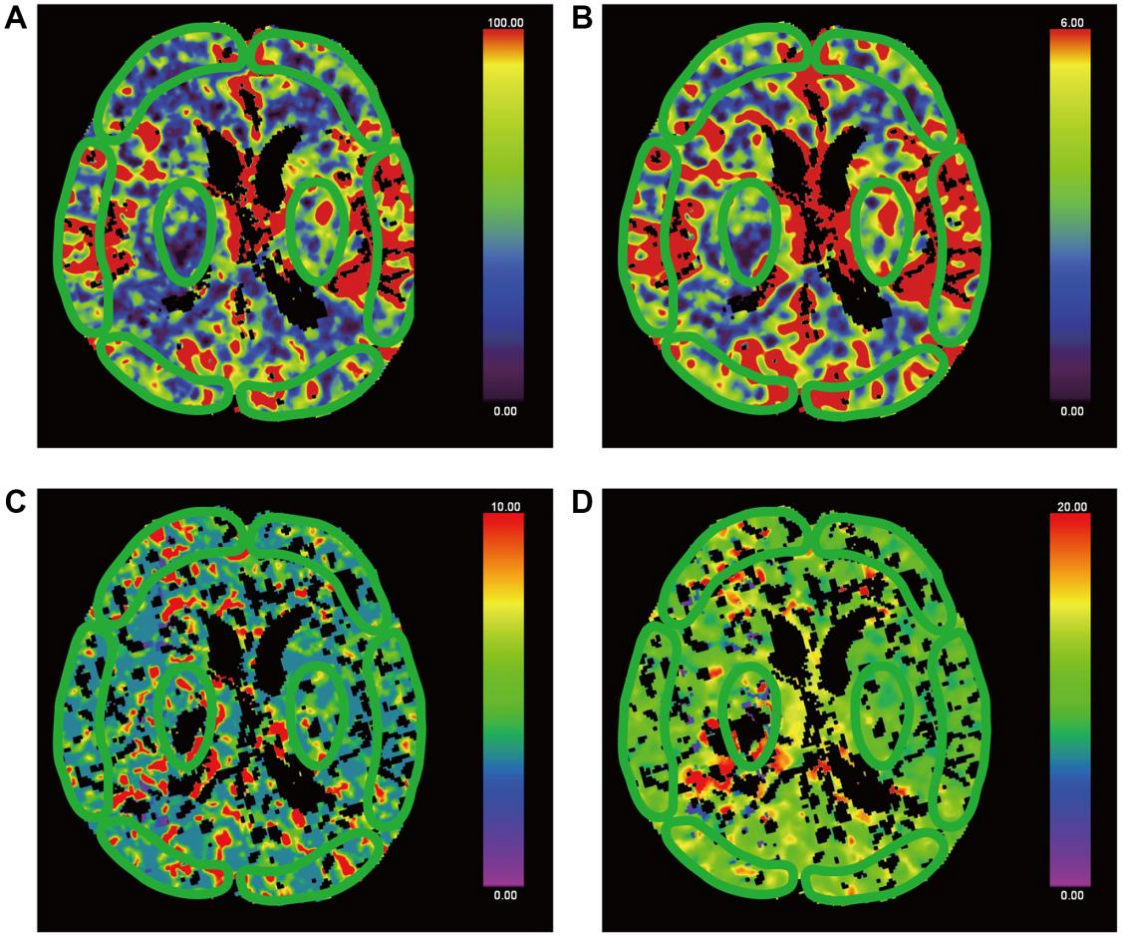
**False-colour pictures of a 76-year-old MCS− man after right thalamus hemorrhage at the basal ganglia level.** The affected side is right. The damaged part is right thalamus. (**A**) The CBF map of the bilateral frontal cortex, temporal cortex, occipital cortex and thalamus, (**B**) the CBV map, (**C**) the TTP map and (**D**) the MTT map.

**Figure 4:**
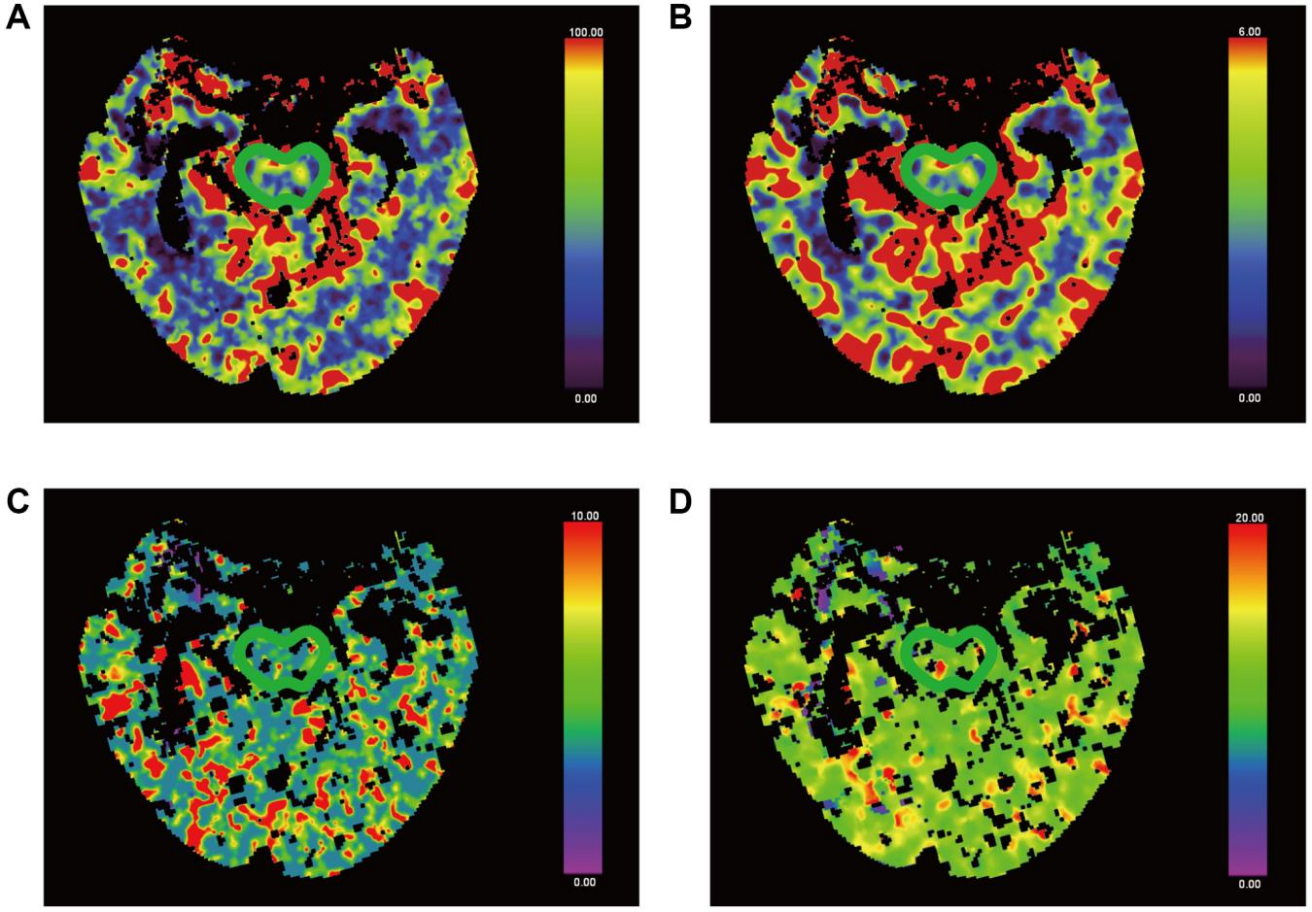
**False-colour pictures of a 76-year-old MCS− man after right thalamus hemorrhage at the pontine level.** The affected side is right. (**A**) The CBF map of the brainstem, (**B**) the CBV map, (**C**) the TTP map and (**D**) the MTT map.

Brain pCT was measured by two clinicians experienced in the imaging diagnosis of neurological disorders. A workstation (Syngo.via, version VA30A; Siemens Healthineers) was used for post-processing. The internal carotid artery was chosen for the inflow artery, and the superior sagittal sinus was used as the outflow vein to generate a time radiodensity curve of the blood. Then, CBF, CBV, MTT and TTP perfusion maps were automatically created. Avoiding the great vessels, the same clinicians were responsible for the selection of regions of interests (Figures 3 and 4) in bilateral gray and white matter in the frontal, occipital, and temporal lobes, as well as in the thalamus, brainstem, the damaged part, and contralateral to the damaged part. Meanwhile, the mean CBF, CBV, TTP and MTT in each ROI were calculated. Absolute values are shown in colour maps (CBF is reported in mL/min/100 g of brain tissue, CBV is reported in mL/100 g of brain tissue, and MTT and TTP are reported in seconds).

### Statistics

Continuous variables were expressed as mean ± standard deviation, and categorical variables were expressed as counts and/or frequencies. According to diagnosis, baseline findings were compared between groups by analysis of variance (ANOVA) for continuous variables and the chi-squared test for categorical variables. Because of several variables departed from normal distribution, spearman’s rank correlation coefficients were used to assess the correlations between behavioral assessment scores, age, pDOC duration, and pCT data. All statistical analyses were performed using SPSS (version 20; IBM, Armonk, NY, USA). A *p*-value < 0.05 was considered statistically significant.
